# Research priorities in pediatric rheumatology: The Childhood Arthritis and Rheumatology Research Alliance (CARRA) consensus

**DOI:** 10.1186/1546-0096-6-5

**Published:** 2008-04-01

**Authors:** Sylvia Ota, Randy Q Cron, Laura E Schanberg, Kathleen O'Neil, Elizabeth D Mellins, Robert C Fuhlbrigge, Brian M Feldman

**Affiliations:** 1 Department of Child Health Evaluative Sciences, The Hospital for Sick Children, Toronto, Canada; 2 Department of Pediatrics, The Children's Hospital of Philadelphia, Philadelphia, USA; 3 Department of Pediatrics, Duke University Medical Center, North Carolina, USA; 4 Division of Pediatric Rheumatology, University of Oklahoma Health Sciences Center, Oklahoma City, USA; 5 Department of Pediatrics, Stanford University, Stanford, USA; 6 Division of Immunology, Children's Hospital-Boston, Boston, USA; 7 Department of Pediatrics, University of Toronto, Toronto, Canada; 8 Department of Health Policy Management and Evaluation, University of Toronto, Toronto, Canada; 9 Department of Public Health Sciences, University of Toronto, Toronto, Canada; 10 Division of Rheumatology, The Hospital for Sick Children, Toronto, Canada

## Abstract

**Background:**

North American pediatric rheumatologists have created an investigator-initiated research network (the Childhood Arthritis and Rheumatology Research Alliance – CARRA) to facilitate multi-centre studies. One of the first projects undertaken by this network was to define, by consensus, research priorities for the group, and if possible a first group-sponsored clinical trial in which all members could participate.

**Methods:**

We determined consensus using the Delphi approach. This approach has been used extensively in health research to reach consensus in large groups. It uses several successive iterations of surveys eliciting ideas and opinions from specialists in the field. Three surveys were designed based on this method and were distributed to members of CARRA to elicit and rank-order research priorities.

**Results:**

A response rate of 87.6% was achieved in the final survey. The most highly ranked research suggestion was to study infliximab treatment of uveitis unresponsive to methotrexate. Other highly ranked suggestions were to study i) the treatment of systemic arthritis with anakinra and ii) the treatment of pediatric systemic lupus erythematosus with mycophenolate mofetil.

**Conclusion:**

The Delphi approach was an effective and practical method to define research priorities in this group. Ongoing discussion and cooperation among pediatric rheumatologists in CARRA and others world-wide will help in developing further research priorities and to facilitate the execution of clinical trials in the future.

## Background

The rare nature of pediatric rheumatology conditions poses challenges in conducting adequately powered trials in single institutions. Recognizing the need for – and the previous successes of – multicentre collaborations, the Childhood Arthritis and Rheumatology Research Alliance (CARRA) was formed in 2002 as an investigator-initiated research network aiming to improve outcomes of children with pediatric rheumatic diseases through high-quality clinical trials and clinical translational research. [1]

As a starting point, a CARRA sub-committee surveyed CARRA members to identify and prioritize research questions. The goal of this project was to identify research priorities for an intervention study addressing a pediatric rheumatology problem that is sufficiently common that centres across the network would have an opportunity to participate, but one that is sufficiently rare that the workload at each institution would not be excessive.

Our chosen consensus method was the Delphi approach [2] which has been applied extensively to develop research priorities in social work [3], acute care [4], infection control and hospital epidemiology [5], health services [6], surgical infection [7], and nursing. [8] This method achieves consensus in a large group setting using successive iterations of questionnaires with controlled feedback. The first questionnaire asks individuals to respond to a broad question. Each subsequent questionnaire is developed based on the responses from the preceding questionnaire and the process is repeated until consensus has been reached or when sufficient information exchange is obtained. [9] This approach offers many benefits. The participants are not required to meet as a group, enabling responses to be submitted from individuals who are far apart geographically. [2] The lack of face-to-face contact ensures participant anonymity which reduces peer pressure that can occur in consensus conferences [2,9].  The Delphi approach also balances the effect of dominant personality types in the determination of the final consensus. [2,9]

The Delphi approach has been widely used in the pediatric rheumatology field, both alone and in combination with the Nominal Group Technique; a structured group-meeting format where meeting participants generate, clarify, and methodically vote for ideas until achieving consensus. [9] These processes have yielded criteria for clinical remission in some categories of juvenile idiopathic arthritis [10], core sets of measures for disease activity and damage in juvenile dermatomyositis and juvenile systemic lupus erythematosus [11], definitions of improvement in juvenile [12] and adult rheumatoid [13] arthritis, definitions of improvement in adult and juvenile myositis, [14] and to develop guidelines for trials of therapies in idiopathic inflammatory myopathies. [15]

The specific aim of this study was to determine pediatric rheumatology research priorities for CARRA using the Delphi technique.

## Methods

The study was carried out between October 2003 and June 2004.

### Study population

Our participants were members of the Childhood Arthritis and Rheumatology Research Alliance (CARRA), representing pediatric rheumatology experts across Canada and the United States.

### Questionnaires

**Figure 1:**
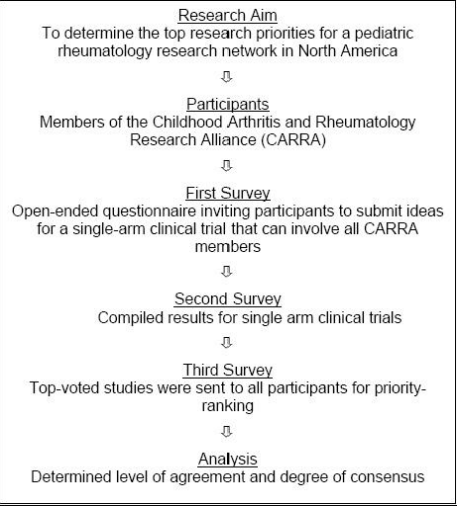
Delphi approach for establishing pediatric rheumatology research priorities.

The Delphi approach is a systematic process used to generate ideas and arrive at consensus in a large group. The first step in our study involved soliciting research ideas using an open-ended questionnaire. These research suggestions were collated and categorized, and a second questionnaire was created with the intent of identifying the most widely accepted suggestions. The most highly endorsed research ideas were sent out in a third questionnaire, to be priority-ranked by the participants (See Figure 1).

#### First questionnaire

The first questionnaire used an open-ended format with two questions: "*What disease or problem should be studied in the first protocol"? *and "*What treatment should be studied for this disease?"*. The questionnaire specified that the proposed study should be a single-arm treatment study of a rare disease or uncommon manifestation of disease, for which a randomized trial would not likely be feasible at the current time. The treatment should be one of interest to the group but not overly controversial so that the majority of CARRA members would feel comfortable enrolling subjects.

Respondents were asked to be as specific as possible and to provide a rationale or justification for their disease of choice and for the recommendation of the therapeutic approach for the study. Participants could respond via email, fax, or postal mail.

#### Second questionnaire

Responses from the first survey were compiled and tallied. Participants were sent a list of suggestions in a new questionnaire and were asked to check off their top ten choices indicating what they would like to see studied across CARRA. They were asked to respond based on the feasibility as a single-arm CARRA supported study, the importance of the research question, and the availability of treatments. Only suggestions for single-arm clinical trials were included.

#### Third questionnaire

In the final questionnaire, the top eight suggestions – 8 were chosen rather than more or fewer based on a natural cut-off in the rankings – from the single-arm clinical trial list were sent to all members, along with a count of the number of top-ten votes that each garnered and the original rationale for the study. Respondents were asked to rank-order each option (with no ties) based on the same criteria as the second questionnaire (feasibility, importance, and availability of treatments). Systemic-onset juvenile rheumatoid arthritis treated with anti-IL-6 receptor antibody was a popular choice in the second questionnaire but the drug would not be available for study in the near future in North America so it was not a viable option for the project and was omitted along with an explanation on the covering letter.

### Survey methods

The Tailored Design Method [16], a method that maximizes the response rate of mail and internet surveys, was used. All correspondence was conducted using electronic mail, including a "pre-notice", that alerted respondents to the forthcoming questionnaire. An initial mail-out was sent soon thereafter with attachments. Approximately 10 days later a reminder email was sent to those who had not responded. Two subsequent reminders were sent approximately 10 days apart. One and a half months after the first mail-out, the research coordinator made follow-up phone calls to all who had not responded after the reminder emails.

### Analysis

No analysis was needed for the first phase; all suggestions were included. Responses were grouped into disease categories for the subsequent questionnaire.

For the second step, all survey responses were double entered into a specially prepared database to ensure keying accuracy. Top-rated suggestions were determined based on natural cut-offs.

For the last questionnaire iteration, all responses were double entered into a computer database. Median rankings and 95% confidence intervals were calculated for each of the eight research suggestions. The Kruskal-Wallis nonparametric analysis of variance was used to compare responses to determine whether the most highly ranked suggestion was indeed statistically significantly different from other suggestions. Statistical calculations used DataDesk 6.2.1 software (Data Description Inc., Ithaca, NY).

## Results

All 107 CARRA members at the time of the study were contacted to participate in the surveys. One member was removed from our mailing list because the e-mail address was not functional and repeated attempts to contact the member were unsuccessful. One other member no longer worked at the institution listed in the CARRA directory and no forwarding address was given. Despite attempts to locate this person, no contact information was found and this person was also removed from the mailing list.

### Iteration 1

Eighty-one out of 105 eligible pediatric rheumatologists (77.1%) responded to the first questionnaire. Many suggestions were made and there were many duplicates. (All suggestions are in Appendix A. See additional file 1: Appendix A: Verbatim Responses for First Delphi Questionnaire). Twenty-four respondents did not submit any suggestions.

### Iteration 2

Eighty-six out of 105 pediatric rheumatologists (81.9%) responded to the second questionnaire. The most highly-endorsed suggestion was to study *systemic-onset juvenile rheumatoid arthritis (JRA) treated with anti-IL 6 *(62 votes), followed by *uveitis (JRA, sarcoid, or idiopathic) unresponsive to methotrexate treated with infliximab *(50 votes), *pediatric systemic lupus erythematosus (SLE) treated with mycophenolate mofetil *(48 votes), and *thrombocytopenia unresponsive to standard therapy in SLE treated with rituximab *(47 votes). A full list of suggested problems and treatments and their number of votes are in Table 1.

**Table 1:** Delphi 2 Responses in order of descending number of votes

**PROBLEM**	**TREATMENT**	**VOTES**
Systemic onset juvenile rheumatoid arthritis	Anti-IL-6 receptor antibody	62
Uveitis (juvenile rheumatoid arthritis, sarcoid, or idiopathic) unresponsive to methotrexate	Infliximab	50
Pediatric systemic lupus erythematosus	Mycophenolate mofetil	48
Thrombocytopenia unresponsive to standard therapy in systemic lupus erythematosus	Rituximab	47
Juvenile dermatomyositis unresponsive to prednisone and disease-modifying anti-rheumatic drugs	Enbrel	43
Systemic onset juvenile rheumatoid arthritis	Thalidomide	43
Systemic onset juvenile rheumatoid arthritis	Anakinra	36
Localized scleroderma	Combination: steroids and methotrexate	35
Juvenile dermatomyositis unresponsive to prednisone and disease-modifying anti-rheumatic drugs	Infliximab	29
Rash of juvenile dermatomyositis	Topical tacrolimus	27
Systemic lupus erythematosus proliferative nephritis unresponsive to pulse cyclophosphamide and mycophenolate mofetil	Combination: pulse cyclophosphamide and mycophenolate mofetil	25
Chronic Recurrent Multifocal Osteomyelitis that is not controlled by non-steroid anti-inflammatory drugs and/or that requires corticosteroids	Bisphosphonates	24
Localized scleroderma	Methotrexate orally	20
Linear scleroderma refractory to oral or subcutaneous methotrexate treatment	Oral mycophenolate mofetil treatment	19
Henoch Scholein Purpura	Glucocorticoid treatment	18
Osteoporosis	Alendronate and other possibilities	18
Pauciarticular juvenile rheumatoid arthritis	Intraarticular steroids	17
Systemic onset juvenile rheumatoid arthritis	Combination:TNF blockers and thalidomide	17
Behcet's	Infliximab	16
Early polyarticular juvenile rheumatoid arthritis	CTLA4-1g	16
Macrophage activation syndrome	Combination: cyclosporin + corticosteroids	15
Sarcoid arthritis	Enbrel	15
Severe systemic lupus erythematosus – cyclophosphamide dependent or resistant	Intravenous fludarabine	15
Rash of systemic lupus erythematosus	Topical tacrolimus	14
Acute rheumatic fever	Naproxen	13
Henoch Scholein Purpura with abdominal pain	Glucocorticoid treatment	13
Juvenile rheumatoid arthritis-temporomandibular joint arthritis	Corticosteroid joint injections	13
Systemic onset juvenile rheumatoid arthritis	Oral intravenous immunoglobulin as adjuvant therapy	10
Familial Mediterranean fever not responsive to colchicine	Anakinra	8
Refractory iritis in juvenile rheumatoid arthritis	Cyclosporin	8
Osteoporosis	Vitamin D – the various preparations	7
Raynaud's 1^0 ^and 2^0^	Biofeedback	7
Familial Mediterranean fever	Anti-Interleukin 1	5
Systemic onset juvenile rheumatoid arthritis	Combination: hydroxychloroquine and standard therapy	2
Systemic onset juvenile rheumatoid arthritis	Combination: thalidomide and cyclosporin	2

### Iteration 3

Ninety-two out of 105 eligible pediatric rheumatologists (87.6%) responded to the third questionnaire. The most highly ranked item was *uveitis (JRA, sarcoid, or idiopathic) unresponsive to methotrexate treated with infliximab*, with a mean ranking score of 2.86 and median ranking of 2 (confidence limits 1, 3 a = 0.05). This item was significantly different from all other items (T = 175.85, p < 0.0001). A complete list of rankings is in Table 2.

**Table 2:** Delphi 3 Responses

**PROBLEM-TREATMENT**	**MEAN**	**MEDIAN**	**CONFIDENCE INTERVAL AROUND MEDIAN (α = 0.05)**	
			
			**Lower Limit**	**Upper Limit**
Uveitis (JRA, sarcoid, or idiopathic) unresponsive to MTX treated with infliximab	2.86	2	1	3
Systemic onset JRA treated with anakinra	3.68	3	3	4
Pediatric SLE treated with MMF	4.15	4	3	5
JDM unresponsive to prednisone and DMARDS treated with etanercept	4.59	4	4	6
Systemic onset JRA treated with thalidomide	4.28	5	3	5
Thrombocytopenia unresponsive to standard therapy in SLE treated with rituximab	4.57	5	4	5
Localized Scleroderma treated with steroids and MTX	4.88	5	4	6
Pauci-articular JRA with uveitis, with early treatment	6.97	8	8	8

JRA: Juvenile Rheumatoid Arthritis

MTX: Methotrexate

SLE: Systemic Lupus Erythematosus

MMF: Mycophenolate mofetil

JDM: Juvenile Dermatomyositis

DMARDS: Disease-Modifying Anti-Rheumatic Drugs

## Discussion

CARRA is a network of pediatric rheumatologists that was formed to answer top-priority questions with potential to improve the care of children with rheumatic diseases. [1] Soon after its establishment, this group surveyed its members to define its research priorities and identified *uveitis (JRA, sarcoid, or idiopathic) unresponsive to methotrexate treated with infliximab *as the top priority for a single-arm treatment trial in this research network. Other top-priority single-arm trials included *systemic onset JRA treated with anakinra *and *pediatric SLE treated with mycophenolate mofetil*. The Delphi approach was a feasible and successful method to identify these research priorities.

To our knowledge, this is the first study of its kind in this subspecialty. Most published literature on pediatric rheumatology priorities focus on enhancing residency programs and the need for expansion of the subspecialty [17-20]. The Pediatric Rheumatology International Trials Organization (PRINTO) has not surveyed its members to determine research priorities on an international level (personal communication, January 16, 2007), and to our knowledge there have been no initiatives in other countries or regions to establish such priorities. A survey of pediatric rheumatologists in North America and Europe was performed to determine types of ongoing trials in the field, to identify any problems encountered in these trials, and willingness to participate in collaborative studies but did not identify actual research topics or priorities[21].

We surveyed all CARRA members and achieved a high response rate; however there are a substantial number of pediatric rheumatologists that are not affiliated with CARRA and are therefore not represented in our study. As it is a North American network, the opinions of those who practice or do research in other continents are also not included. Pediatric rheumatology research priorities in North America may differ substantially from those in Europe or the developing world due to differences in availability of medications, access to health care, referral patterns, and cultural acceptability of treatment, and thus our survey results represent only the opinions of a subset of North American pediatric rheumatologists.

CARRA is a large organization that, at the time of our survey had 107 members and has now grown to 171 members. Members are typically interested in research and represent a sizable proportion of pediatric rheumatologists in North America. There were 34 physicians taking care of children with rheumatic diseases in Canada in the year this survey was conducted (personal communication, August 8, 2007) of which nine were CARRA members; and there were an estimated 192 to 215 board certified pediatric rheumatologists in the United States (personal communication, American Board of Pediatrics, August 8, 2007) of which 92 were contacted for this survey. Over 40% of those treating children with rheumatic diseases were therefore represented in our survey. Six additional CARRA members are sponsored members with PhD degrees with expertise in pediatric rheumatology. With our high response rate, we are confident that our results are reasonably representative of those pediatric rheumatologists in North America with research interests.

The process that was undertaken by this group has resulted in the identification of important research ideas, with the two most highly-endorsed topics currently moving forward for scientific investigation. A request-for-proposals was put forth to investigate the top-rated suggestion of infliximab treatment in patients with persistent uveitis, and a CARRA-funded study is currently underway. A proposal for investigating the treatment effect of IL-1 inhibition in patients with systemic-onset juvenile arthritis has also been developed by CARRA investigators and has secured funding.

## Conclusion

In summary, the Delphi method was successfully used to identify pediatric rheumatology research priorities in North America. The top-priority for a single-arm trial was identified as a study for uveitis patients (JRA, sarcoid, or idiopathic) unresponsive to methotrexate, treated with infliximab. This provides a starting point for further research and clinical trials in pediatric rheumatology.

## Competing interests

The author(s) declare that they have no competing interests.

## Authors' contributions

SO coordinated the data collection, performed the analysis and interpretation of the data, and drafted the manuscript. RC, LS, KO, EM, and RF were involved in the conception and design of the study and critical revision of the manuscript. BF was involved in the conception and design of the study, data collection, analysis and interpretation of the data, and critical revision of the manuscript. All authors read and approved the final manuscript.

## Supplementary Material

Additional file 1 Appendix A: Verbatim Responses for First Delphi Questionnaire. Responses to first questionnaire as provided by respondents. Click here for file
