# Supplementary material for: Research priorities in pediatric rheumatology: The Childhood Arthritis and Rheumatology Research Alliance (CARRA) consensus
Source: Pediatr Rheumatol Online J. 2008 Apr 1;6:5. doi: 10.1186/1546-0096-6-5 (PMC2330140; doi:10.1186/1546-0096-6-5)
Supplement: Additional file 1 — Appendix A: Verbatim Responses for First Delphi Questionnaire. Responses to first questionnaire as provided by respondents. [file 1546-0096-6-5-S1.pdf]

Appendix A: Verbatim Responses for First Delphi Questionnaire

DISEASE/MANIFESTATION	TREATMENT
**Juvenile Idiopathic Arthritis**	
Systemic onset JIA	- No treatment suggested<br>- Thalidomide<br>- TNF blockers and thalidomide<br>- Hydroxychloroquine in combination with standard therapy<br>- Oral IVIG as adjuvant therapy<br>- Anakinra<br>- Anti-IL-6 receptor antibody<br>- Standard protocols depending on disease activity<br>- Thalidomide and cyclosporin
JIA temporomandibular joint arthritis	- Corticosteroid joint injections
Pauciarticular juvenile rheumatoid arthritis	- Intraarticular steroids for therapy
Early polyarticular juvenile rheumatoid arthritis	- CTLA4-Ig
Juvenile rheumatoid arthritis unresponsive to disease-modifying	- DMARDS - Expand indications and label changes
JIA	- Silymarin
Lipid profiles in JIA	- Patients on TNF inhibitors compared to those children not on TNF inhibitors
**Juvenile Dermatomyositis (JDM)**	
JDM	- Multi-institutional, mechanism-focused study<br>- Suggestion IV pulse steroid with oral steroids versus suggestion IV pulse steroid with oral steroids + methotrexate
JDM unresponsive to prednisone and DMARDs	- Enbrel/Infliximab
Calcinosis in JDM	- Bisphosphonates vs. diltiazem<br>- Diltiazem, pamidronate, or immunosuppressive approaches, with solid outcome measures<br>- Therapy with diltiazem + alendronate versus diltiazem alone
**Henoch Schonlein Purpura**	
Henoch Schonlein Purpura	- Glucocorticoid treatment<br>- Steroids early
Henoch Schonlein Purpura with abdominal angina	- Short course steroid therapy
**Systemic Lupus Erythematosus (SLE)**	
SLE proliferative nephritis unresponsive to pulse cyclophosphamide and mycophenolate mofetil	- Combination pulse cyclophosphamide and mycophenolate mofetil
Pediatric SLE	- Mycophenolate mofetil
Severe SLE<br>1. Who are good or partial responders to cyclophosphamide but acquired high cumulative doses of cyclophosphamide over the years, or 2. who are poor responders to cyclophosphamide, or 3. who are high risk for adverse effects of cyclophosphamide	- Intravenous fludarabine as a cyclophosphamide sparing agent
SLE patients with antiphospholipid antibodies	- ASA vs ASA/warfarin for the prevention of thromboembolic events

Thrombocytopenia unresponsive to standard therapy in SLE	- Rituximab infusions versus conventional therapy.
**SCLERODERMA**	
Scleroderma	- Steroids and methotrexate<br>- Cyclophosphamide vs. Bone Marrow Transplant (with PRES)
Localized scleroderma	- Methotrexate vs. placebo<br>- Methotrexate vs. prednisone<br>- Methotrexate vs. penicillamine with or without steroids<br>- Methotrexate orally
Morphea and linear scleroderma – new onset	- Methotrexate weekly<br>- Methotrexate orally<br>- Methotrexate
Linear scleroderma refractory to oral or subcutaneous methotrexate treatment.	- Oral mycophenolate mofetil
**IMMUNOGENICITY**	
Immunization of children on biologics or immunosuppressive medication	- Physiotherapy approaches: Significance of correcting leg length discrepancy<br>- Efficacy of MMF in childhood lupus
The immunogenicity of hepatitis B vaccine in pediatric patients with rheumatic diseases on immunosuppressive medications	- No treatment
**UVEITIS (Iritis)**	
Uveitis (JRA, sarcoid, or idiopathic) not responsive to methotrexate	- Infliximab
JRA uveitis unresponsive to methotrexate	- Infliximab
Methotrexate-resistant uveitis	- Infliximab
Uveitis	- Multicentre Study
Pauciarticular JRA with associated uveitis within the first six months of diagnosis	- Early induction treatment
Resistant or recurrent uveitis in pauciarticular JIA.	- Infliximab
Iritis associated with JRA and idiopathic iritis unresponsive to methotrexate and/or cyclosporin	- Infliximab
Refractory iritis in JRA	- Cyclosporin
**OTHER TYPES**	
EBV lymphoproliferative disease	- No treatment suggested
Arthritis of Blau syndrome	- No treatment suggested
Sarcoid arthritis	- Enbrel
Acute rheumatic fever	- Naproxen
Lupus nephritis in children and the use of immunosuppressive therapy.	- Send survey, gather info, develop a registry
Idiopathic retro-orbital pseudotumor	- Combination therapy: pulse corticosteroid + weekly methotrexate +/- cyclosporin
Behcet disease	- Infliximab
Familial Mediterranean fever not responsive to colchicine	- Anakinra
Chronic recurrent multifocal Osteomyelitis (CRMO) that is not controlled by	- Bisphosphonates

NSAIDs and/or that requires corticosteroids	
CRMO/SAPHO (Synovitis, Acne, Pustulosis, Hyperostosis, Osteitis)/DSOM (Diffuse Sclerosing Osteomyelitis of the Mandible)	- Bisphosphonates in children
Rash of SLE or JDM	- Topical tacrolimus
Raynaud's 1^0 and 2^0	- Biofeedback
Macrophage activation syndrome	- Cyclosporin +/- corticosteroids
What is the rate of bone fracture in children with :<br>a) SLE, JRA, JDM prior to therapy<br>b) The above diseases, after immunosuppressive therapy (0-5 years) controlling for all medications/supplements<br>c) Are the rates of bone fracture different?	- Vitamin D--the various preparations<br>- Fosamax and other possibilities
Consensus conference for standard of care of a few diseases.	
Study a very rare disease by characterizing it better clinically and trying to understand the pathogenic mechanisms better by collecting biologic samples (e.g. macrophage activation syndrome)	

JRA: Juvenile Rheumatoid Arthritis
JIA: Juvenile Idiopathic Arthritis
MTX: Methotrexate
SLE: Systemic Lupus Erythematosus
MMF: mycophenolate mofetil
JDM: Juvenile Dermatomyositis
DMARDs: Disease-Modifying Anti-Rheumatic Drugs
NSAIDs: Non-Steroidal Anti-Inflammatory Drugs
IVIG: Intravenous Immunoglobulin
PRES: Pediatric Rheumatology European Society
ASA: Acetylsalicylic Acid
TNF: Tumor Necrosis Factor
